# Hypoxia Promotes Osteogenesis but Suppresses Adipogenesis of Human Mesenchymal Stromal Cells in a Hypoxia-Inducible Factor-1 Dependent Manner

**DOI:** 10.1371/journal.pone.0046483

**Published:** 2012-09-27

**Authors:** Markus Wagegg, Timo Gaber, Ferenz L. Lohanatha, Martin Hahne, Cindy Strehl, Monique Fangradt, Cam Loan Tran, Kerstin Schönbeck, Paula Hoff, Andrea Ode, Carsten Perka, Georg N. Duda, Frank Buttgereit

**Affiliations:** 1 Department of Rheumatology and Clinical Immunology, Charité University Hospital, Berlin, Germany; 2 German Arthritis Research Center, Berlin, Germany; 3 Berlin-Brandenburg Center of Regenerative Therapies, Charité University Hospital, Berlin, Germany; 4 Julius Wolff Institute and Center for Musculoskeletal Surgery, Charité University Hospital, Berlin, Germany; 5 Orthopaedic Departments, Charité University Hospital, Berlin, Germany; 6 Berlin-Brandenburg School of Regenerative Therapies, Charité University Hospital, Berlin, Germany; University of Sao Paulo - USP, Brazil

## Abstract

**Background:**

Bone fracture initiates a series of cellular and molecular events including the expression of hypoxia-inducible factor (HIF)-1. HIF-1 is known to facilitate recruitment and differentiation of multipotent human mesenchymal stromal cells (hMSC). Therefore, we analyzed the impact of hypoxia and HIF-1 on the competitive differentiation potential of hMSCs towards adipogenic and osteogenic lineages.

**Methodology/Principal Findings:**

Bone marrow derived primary hMSCs cultured for 2 weeks either under normoxic (app. 18% O_2_) or hypoxic (less than 2% O_2_) conditions were analyzed for the expression of MSC surface markers and for expression of the genes *HIF1A*, *VEGFA*, *LDHA*, *PGK1*, and *GLUT1*. Using conditioned medium, adipogenic or osteogenic differentiation as verified by Oil-Red-O or von-Kossa staining was induced in hMSCs under either normoxic or hypoxic conditions. The expression of *HIF1A* and *VEGFA* was measured by qPCR. A knockdown of HIF-1α by lentiviral transduction was performed, and the ability of the transduced hMSCs to differentiate into adipogenic and osteogenic lineages was analyzed. Hypoxia induced HIF-1α and HIF-1 target gene expression, but did not alter MSC phenotype or surface marker expression. Hypoxia (i) suppressed adipogenesis and associated *HIF1A* and *PPARG* gene expression in hMSCs and (ii) enhanced osteogenesis and associated *HIF1A* and *RUNX2* gene expression. shRNA-mediated knockdown of HIF-1α enhanced adipogenesis under both normoxia and hypoxia, and suppressed hypoxia-induced osteogenesis.

**Conclusions/Significance:**

Hypoxia promotes osteogenesis but suppresses adipogenesis of human MSCs in a competitive and HIF-1-dependent manner. We therefore conclude that the effects of hypoxia are crucial for effective bone healing, which may potentially lead to the development of novel therapeutic approaches.

## Introduction

Mesenchymal stromal cells (MSCs) are a pluripotent cell population capable of differentiating into a variety of cell types including osteoblasts, chondrocytes, adipocytes, and myoblasts [1]. MSCs are essential for the repair and regeneration of damaged tissues, and can be easily isolated from numerous tissues [2], [3]. Therefore, cell therapy using MSCs represents a promising approach to promote wound healing and tissue regeneration, such as in repair of bone fractures.

Bone healing is characterized by a series of cellular and molecular events that commence with hematoma formation and an inflammatory cascade, finally leading to MSC recruitment and terminal MSC differentiation. MSC recruitment is known to be essential for successful fracture repair, and recent studies have shown that migration of MSCs is strongly influenced by mechanical stimulation equivalent to conditions of the early bone-healing phase [4].

This process takes place under low O_2_ tensions – so called hypoxia – which is mainly due to the disruption of supplying blood vessels [5]. When comparing with atmospheric oxygen levels (21% O_2_) or *in vitro* culture condition (∼18% O_2_), *in vivo* physiological O_2_ tension of peripheral tissues, e.g. in the stem cell niches such as adipose tissue or bone marrow is much lower (1–7% O_2_) (as reviewed in [6]).

One key event in the cellular adaptation towards a hypoxic environment is the induction/stabilization of the transcription factor hypoxia-inducible factor (HIF)-1, which is composed of an oxygen-sensitive α-subunit and a constitutively expressed β-subunit. In the presence of higher oxygen levels (>5%), HIF-1α protein is subjected to proteosomal degradation almost as soon as it is translated. Under hypoxic conditions, HIF-1α protein is stabilized, dimerizes with HIF-1β and transactivates a number of genes whose products participate in a variety of cellular processes involved in adaptation to hypoxia, such as glycolysis, erythropoiesis, and angiogenesis [7], [8].

Several studies have been carried out in order to analyze the effects of hypoxia on MSCs, but the results were either inconsistent or yielded conflicting results. For example, some reports demonstrated that human bone marrow-derived MSCs cultured under hypoxia showed a diminished capacity to differentiate into adipocytes and osteocytes, supporting the notion that low oxygen tension promotes an undifferentiated state [9], [10], [11]. In contrast, other reports demonstrated that MSCs expanded under reduced oxygen tension to be primed for chondrogenic differentiation [12], [13]. Moreover, Tsai et al recently demonstrated that (i) hypoxic culture conditions promote chondrogenic, osteogenic, and adipogenic differentiation and (ii) hypoxic cells show an increased bone repairing ability *in vivo* [14].

In summary, although the observations are highly variable depending on the experimental conditions and specimens used, it is clear that hypoxia (and hence HIF-1α) affects the differentiation characteristics of MSCs. However, much less attention has been paid to the role of oxygen as a signalling molecule that influences human stromal cell survival, proliferation and differentiation in culture. Only limited and inconsistent information is available on the impact of hypoxia and HIF-1α on the differentiation potential of primary human multipotent MSCs. Information on these crucial issues are, however, highly clinically relevant. Therefore, we investigated the effect of hypoxia on the differentiation potential of primary human MSCs.

## Results

### Bone marrow derived human MSCs express typical surface markers and are able to differentiate into adipogenic, osteogenic and chondrogenic lineages

**Figure 1:**
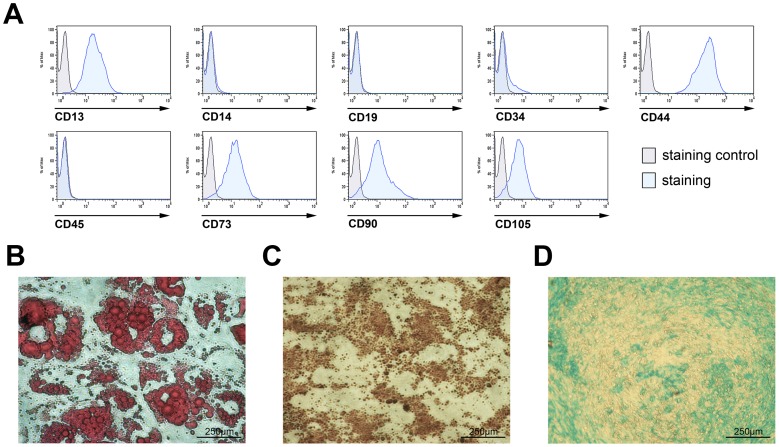
Characterization of human MSCs by their surface marker expression and their ability to differentiate into mesenchymal lineages. (**a**) Bone marrow derived human MSCs express CD13, CD44, CD73, CD90, and CD105 but do not express CD45, CD34, CD14, and CD19; they differentiate into different mesenchymal lineages such as (**b**) adipogenic, (**c**) osteogenic and (**d**) chondrogenic lineages (scale indicated on the figures; n = 8).

Isolated bone marrow derived human MSCs were characterized via their surface marker expression according to the declaration of the *Mesenchymal and Tissue Stem Cell Committee of the International Society for Cellular Therapy*, being positive for the expression of CD13, CD44, CD73, CD90, and CD105 and negative for the expression of CD45, CD34, CD14, and CD19 (**Fig. 1a** **,** **Tab. 1**). The MSC phenotype was confirmed by showing differentiation into different mesenchymal lineages such as adipogenic, chondrogenic and osteogenic (**Fig. 1b–d**).

**Table 1:** Percentage of CD-positive cell populations.

Patient#	CD13	CD14	CD19	CD34	CD44	CD45	CD73	CD90	CD105
**1**	99.5	0.1	0.5	2.1	95.1	0.1	100.0	95.0	87.5
**2**	99.9	0.2	0.8	1.2	96.2	0.2	100.0	93.2	86.1
**3**	99.8	0.5	0.4	0.4	91.8	0.0	100.0	94.1	89.9
**4**	99.2	1.2	0.7	0.6	96.5	0.2	100.0	93.2	93.4
**5**	98.7	0.3	0.1	3.5	99.0	0.1	100.0	91.1	85.2
**6**	98.2	0.6	0.7	2.0	90.1	0.3	100.0	92.1	92.2
**7**	99.5	0.4	0.5	4.1	92.4	0.1	100.0	94.3	96.1
**9**	99.3	1.1	0.2	0.6	91.6	0.1	100.0	95.2	81.7
**mean**	**99.1**	**0.7**	**0.4**	**1.9**	**93.6**	**0.1**	**100.0**	**93.3**	**89.8**
**SD**	**0.6**	**0.4**	**0.3**	**1.6**	**3.4**	**0.1**	**0.0**	**1.5**	**5.4**

### Hypoxia does not alter MSC phenotype and surface marker expression

**Figure 2:**
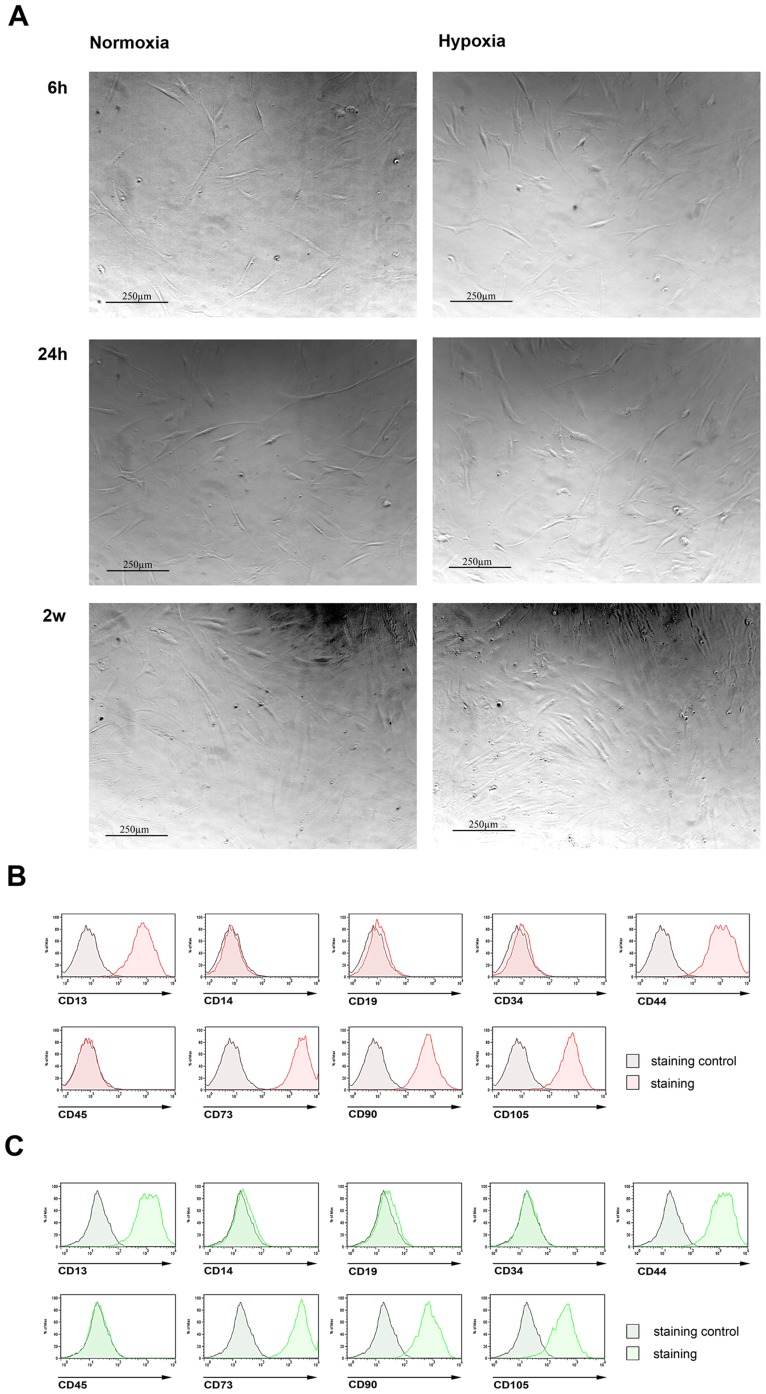
Hypoxia does not alter MSC phenotype and surface marker expression. No difference between MCSs maintained under normoxia and those incubated under hypoxia were observed in terms of (**a**) cell morphology (after 6 h, 24 h, and 2w of incubation; scale indicated on the figures; n = 3) and (**b**) surface marker expression of the positive-markers CD13, CD44, CD73, CD90, CD105 and the negative-markers CD45, CD34, CD14, and CD19 (normoxia = red; hypoxia = green; n = 6).

In order to analyze the impact of hypoxia on human MSC phenotype and surface marker expression, isolated bone marrow derived cells were cultured in DMEM without growth or differentiation factors supplemented with 10% FCS. MSCs were cultured for 2 weeks under normoxic and hypoxic conditions. We found no difference in cell morphology between MCSs maintained under hypoxia and those incubated under normoxia for periods of 6 hours, 24 hours or 2 weeks (**Fig. 2a**). Subsequently, immunostaining analysis of surface markers of MSCs cultured under normoxic or hypoxic conditions was performed. Under both conditions, the surface markers CD13, CD44, CD73, CD90, and CD105 were expressed whereas markers CD45, CD34, CD14, and CD19 were absent without any detectable differences between normoxic and hypoxic cell culture conditions (**Fig. 2b** **,** **Tab. 2** **and** **3**).

**Table 2:** Percentage of CD-positive cell populations after 2 week incubation under normoxia.

Patient#	CD13	CD14	CD19	CD34	CD44	CD45	CD73	CD90	CD105
**3**	98.7	1.2	0.2	2.3	97.5	0.0	100.0	99.8	81.8
**4**	91.2	1.1	1.3	1.4	98.2	1.2	100.0	99.2	89.4
**5**	99.3	0.3	0.5	2.2	99.8	0.3	100.0	97.2	90.8
**6**	92.4	0.6	1.2	1.0	98.7	0.2	100.0	99.9	89.0
**7**	92.3	0.4	0.4	1.3	99.5	0.0	100.0	99.0	95.1
**9**	98.1	1.1	0.9	1.2	98.5	1.1	100.0	99.3	95.2
**mean**	**95.3**	**0.8**	**0.8**	**1.6**	**98.7**	**0.5**	**100.0**	**99.3**	**90.2**
**SD**	**3.7**	**0.4**	**0.5**	**0.5**	**0.8**	**0.5**	**0.0**	**1.1**	**4.9**

**Table 3:** Percentage of CD-positive cell populations after 2 week incubation under hypoxia.

Patient#	CD13	CD14	CD19	CD34	CD44	CD45	CD73	CD90	CD105
**3**	99.3	1.6	0.9	1.9	99.3	0.2	100.0	99.5	82.5
**4**	90.9	0.2	2.3	1.9	96.2	0.4	100.0	98.7	88.4
**5**	98.7	0.0	0.7	1.4	98.2	1.3	100.0	94.5	87.6
**6**	99.3	1.4	0.2	2.1	99.0	0.9	100.0	98.7	92.4
**7**	94.8	1.3	0.0	2.3	97.6	0.0	100.0	99.3	93.5
**9**	97.5	0.8	1.7	1.6	98.8	2.3	100.0	98.2	96.8
**mean**	**96.8**	**0.9**	**1.0**	**1.9**	**98.2**	**0.9**	**100.0**	**98.2**	**90.2**
**SD**	**3.3**	**0.7**	**0.9**	**0.3**	**1.1**	**0.9**	**0.0**	**1.8**	**5.1**

### Hypoxia induces HIF-1α and HIF-1 target gene expression

**Figure 3:**
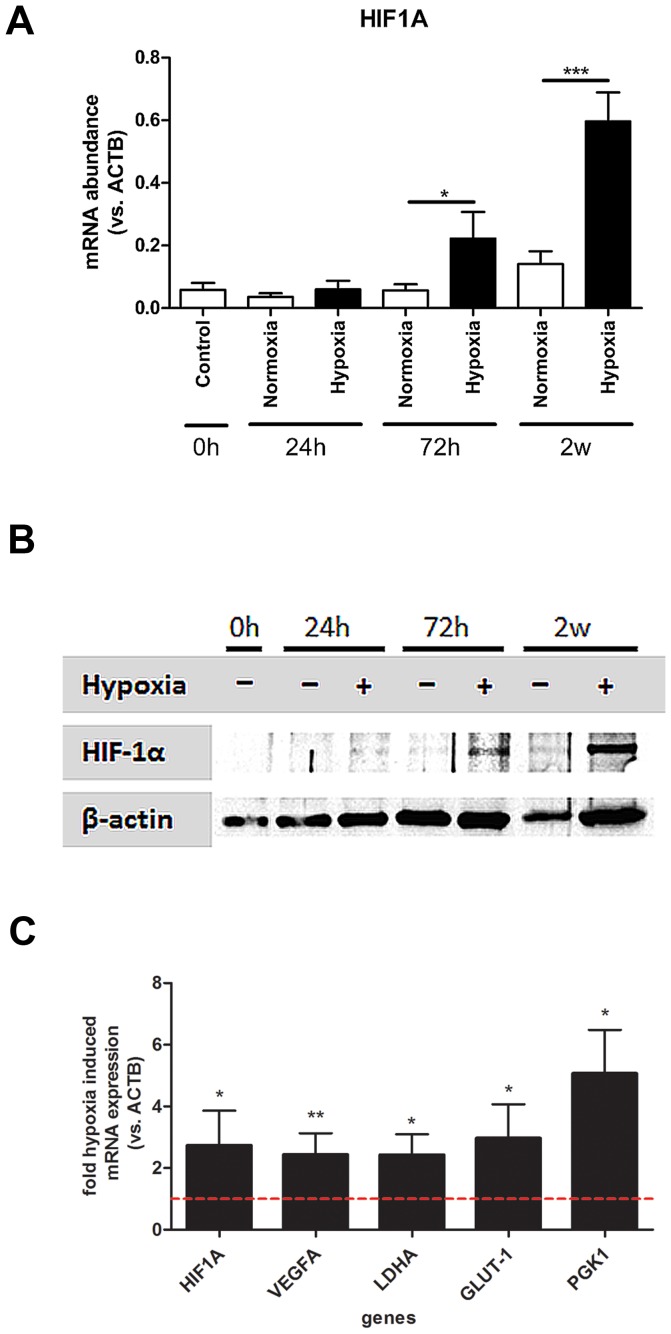
Hypoxia induces HIF-1α and HIF-1-target-gene expression. (**a**) HIF1A gene expression of human MSCs obtained by real-time PCR (n = 6; unpaired t-test). (**b**) HIF-1alpha and beta-actin protein expression obtained by immunoblot. (**c**) Hypoxia-induced HIF-1-target-gene expression of VEGFA, LDHA, GLUT1, and PGK1 (n = 6; 2-weeks data; one sample t-test; dotted-line indicates normalization to gene expression of normoxic cells; *** p<0.001; ** p<0.01; * p<0.05).

Next, we analyzed in MSC the influence of hypoxia on the transcription of HIF-1α. We found hypoxia resulted in a significant time-dependent increase in the expression of HIF-1α mRNA (*HIF1A*) in MSCs after 72 hours (p<0.05) and 2 weeks (p<0.001) of incubation when compared to cells under normoxia (**Fig. 3a**). HIF-1α is known to be primarily regulated at the protein/post-translational level. Therefore, we also investigated the protein expression of HIF-1α and found a time-dependent increase in HIF-1α protein expression (**Fig. 2b**). Furthermore, typical HIF target genes such as *VEGF*, *GLUT1*, *LDHA*, *PGK1* were up-regulated after 72 hours under hypoxic conditions (**Fig. 3c**). The observed increase of HIF-1α and HIF-1-target-gene expression in primary human MSCs suggest that HIF-1α in MSCs is a key regulator for adaption to hypoxia.

### Hypoxia suppresses adipogenic and promotes osteogenic differentiation of human MSCs

**Figure 4:**
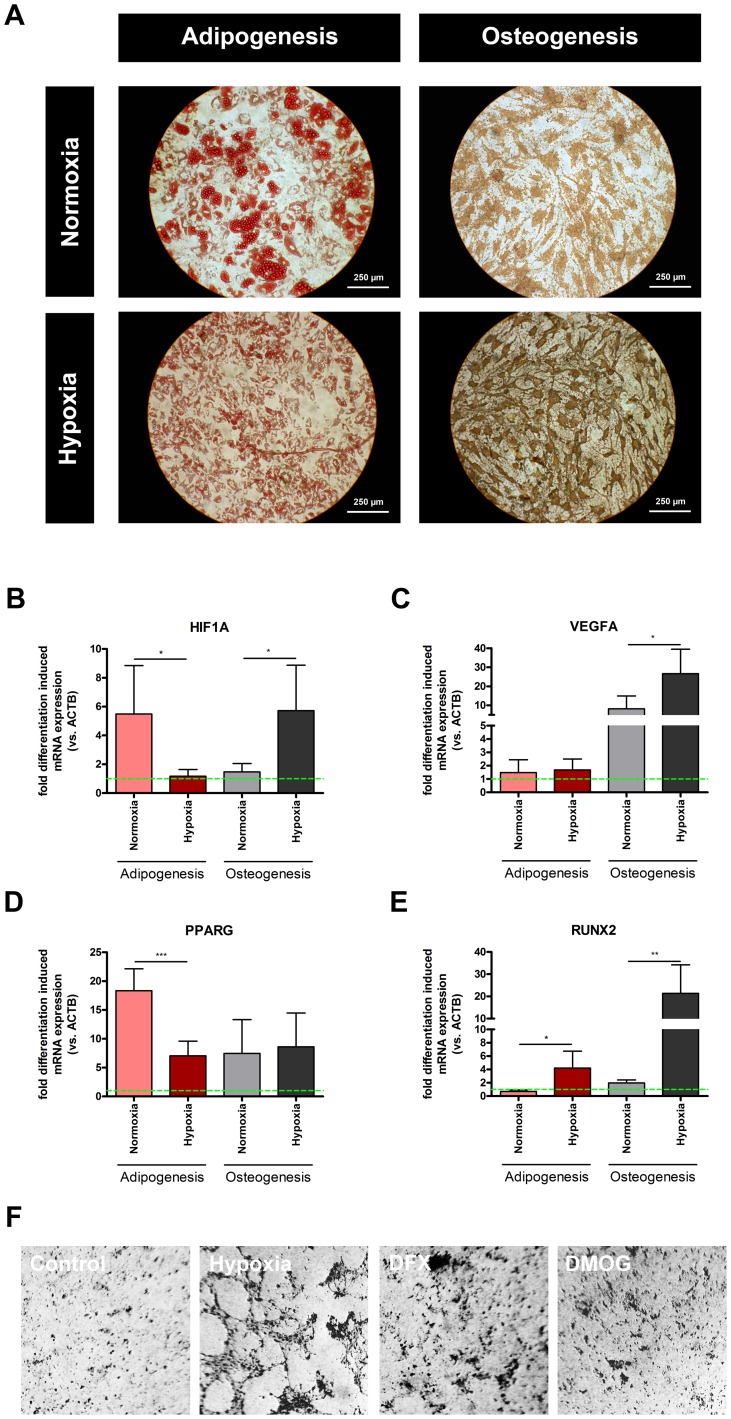
Hypoxia suppresses adipogenic and promotes osteogenic differentiation of human MSCs. (**a**) Oil-Red-O stain for the analysis of adipogenesis and von-Kossa stain for the analysis of osteogenesis of MSCs incubated under normoxia (≈18% pO_2_) or hypoxia (1% pO_2_) for 4 weeks using either osteogenic or adipogenic differentiation medium (scale indicated on the figures; n = 6). (**b**) HIF1A, (**c**) VEGFA, (**d**) PPARG and (**e**) RUNX2 gene expression of MSCs incubated under normoxia (≈18% pO_2_) or hypoxia (1% pO_2_) for 2 weeks using either osteogenic or adipogenic differentiation medium as obtained by real-time PCR (n = 6; unpaired t-test; dotted-line indicates normalisation to gene expression of undifferentiated cells; * p<0.05). (**f**) Analysis of osteogenesis by von-Kossa stain of MSCs incubated under normoxia (≈18% pO_2_) without treatment, or with either 250 µM DFX and 100 µM DMOG, respectively (scale indicated on the figures; n = 3).

From the results above and the reports that hypoxia promotes chondrogenesis [13], we suspected hypoxia may also influence adipogenesis and osteogenesis. In order to test this hypothesis, we induced differentiation of human MSCs into adipocytes and osteoblasts under normoxic and hypoxic conditions, respectively. We found adipogenic differentiation to be suppressed under hypoxia, but osteogenic differentiation to be clearly promoted (**Fig. 4a**). We also analyzed mRNA expression of *HIF1A* and *VEGFA* during adipogenesis and osteogenesis (**Fig. 4b and c**). In the case of adipogenesis and osteogenesis, *HIF1A* mRNA is differentially expressed in MSCs incubated under normoxic and hypoxic conditions (**Fig. 4d**). In adipogenesis, the expression significantly decreases after 2 weeks under hypoxia compared to normoxia (p<0.05). In contrast, during osteogenesis there is an increase of expressed *HIF1A* mRNA under hypoxic conditions compared to normoxia (p<0.05). Surprisingly, expression of *VEGFA* transcription is up to 20 fold higher under hypoxic conditions during osteogenesis than during adipogenesis. It increases with time and is higher under hypoxia than under normoxia (p<0.05). In contrast, *VEGFA* mRNA expression is decreased during adipogenesis (**Fig. 4c**). Furthermore, we analysed the expression of *PPARG* (a key marker for the adipogenic switch), and *RUNX2* (a key marker for the osteogenic switch). In adipogenesis the expression of *PPARG* is significantly higher after 2 weeks under normoxia compared to hypoxia (p<0.001; **Fig. 4d**). In the case of *RUNX2*, we observed a significant up-regulation of gene expression after 2 weeks under hypoxia compared to normoxia which is much more pronounced during osteogenic differentiation (p<0.01) than during adipogenic differentiation (p<0.05; **Fig. 4e**).

We found that osteogenesis of human MSC was facilitated by chemical inducers of HIF-1α, such as the iron chelating agent desferrioxamine mesilate (DFX) or the dimethyloxallyl glycine (DMOG), even under normoxic conditions, but to a lesser extent than under hypoxic conditions (**Fig. 4f**).

### Reduction of HIF-1α expression in human MSCs (i) enhances adipogenesis under normoxic conditions, (ii) partially restores hypoxia-induced attenuation of adipogenesis and (iii) suppresses hypoxia-enhanced osteogenesis

**Figure 5:**
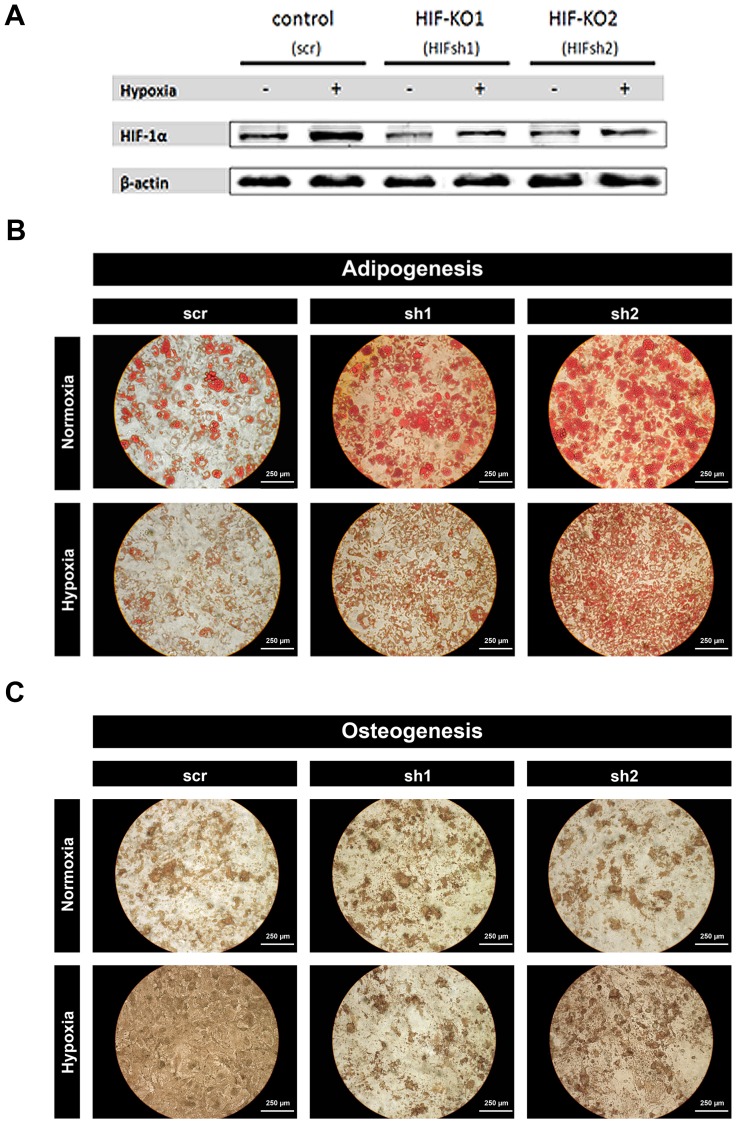
Reduction of HIF-1α of human MSCs (i) enhances adipogenesis under normoxic conditions, (ii) partially restores hypoxia-induced attenuation of adipogenesis and (iii) suppresses hypoxia-enhanced osteogenesis. (**a**) Transduction of anti HIF-1α-shRNA-constructs efficiently reduced HIF-1α protein expression as shown by immunoblot (2-weeks data). (**b**) Oil-Red-O stain for the analysis of adipogenesis of shRNA-construct transduced MSCs and (**c**) von-Kossa stain for the analysis of osteogenesis of shRNA-construct transduced MSCs. Cells were maintained under normoxia (≈18% pO_2_) or hypoxia (1% pO_2_) for 4 weeks using either osteogenic or adipogenic differentiation medium (scale indicated on the figure; n = 3).

From the results above, we considered that HIF-1α could play a key role in regulating adipogenesis and osteogenesis. In order to investigate the function of HIF-1α in greater detail, a shRNA mediated knockdown of HIF-1α was performed using lentiviral transduction. To this end, we used two HIF-1α-silencing shRNAs (sh1 and sh2) and one non-silencing shRNA (scr) as control. Efficiency of lentiviral transduction was up to 93% (data not shown). Knockdown of HIF-1α was verified at the protein level (**Fig. 5a**).

In HIF-1α knockdown cells, adipogenesis and osteogenesis were induced for 4 weeks. In the case of adipogenesis (**Fig. 5b**), control cells (scr-treated cells) behaved as expected, i.e. adipogenesis was suppressed under hypoxia. However, adipogenic differentiation of transduced MSCs was increased compared with controls, under both normoxic and hypoxic conditions.

Interesting

ly, osteogenic differentiation showed the converse (**Fig. 5c**), with promotion of osteogenesis under hypoxic conditions. In the case of osteogenic differentiation of MSCs with HIF-1α knockdown, we found suppression of hypoxia-enhanced osteogenesis.

## Discussion

Mesenchymal stromal cells (MSCs) are pluripotent cells, capable of differentiating into a variety of cell types and being essential in the repair and regeneration of damaged tissues [1]. To this end, MSCs are known to be recruited to sites of injury and inflammation for repair and regeneration of damaged tissue such as injured muscles, tendons and bones where they face conditions of reduced oxygen availability [6], [15]. Hypoxia, and hence HIF-1α, affects (i) MSC maintenance and self-renewal in the stem cell niche under physiological hypoxia and (ii) MSC differentiation in the process of tissue regeneration after injury under pathophysiological hypoxia [3]. Therefore, we investigated the effect of hypoxia on both maintenance and differentiation potential of primary human MSCs.

To this end, we first confirmed the immunophenotype and validated the function of the MSCs isolated in our experiments (**Fig. 1**). We then showed that incubation under hypoxia for up to 2 weeks did not alter the morphology or immunophenotype of human MSCs (**Fig. 2**). Furthermore, we demonstrated the capability of human MSCs to induce HIF-1α and HIF-1 target gene expression under hypoxic conditions (**Fig. 3**). The data demonstrate both the maintenance of the stem cell capabilities, indicated by morphology and immunophenotype, and the switch from aerobic to anaerobic cell metabolism such as postulated for the stem cell niche where the physiological oxygen tension is known to be as low as 1–2% [3].

Moreover, MSCs also face pathophysiologic hypoxia. For example, low oxygen levels are strongly associated with injury and/or inflammation. Under these circumstances the microenvironment is also characterized by cell accumulation/infiltration, and high levels of humoral factors such as cytokines and growth factors.

An interesting and typical example is bone fracture repair [16], [17], [18]. Here, a series of cellular and molecular events is initiated leading to structural reconstitution and tissue regeneration [19]. The bone healing process has been characterized by four phases: an inflammatory phase, a soft callus phase, a hard callus phase, and a remodelling phase [20], [21]. MSCs are thought to be recruited to the fracture site in the initial stages of fracture healing (day 1) and to proliferate by day 3 after fracture [5], [22]. During these stages, MSCs that are utilized to regenerate bone tissue do not only face hypoxic conditions but also high levels of pro-inflammatory mediators [23], [24]. Both factors are considered capable of modifying behaviour and functionality of MSCs.

Given this background, we investigated the differentiation potential of human MSCs towards adipogenic and osteogenic lineages. We found that adipogenesis – although induced with adipogenic conditioned medium – was suppressed by hypoxia, whereas osteogenesis was enhanced, which directly correlated to the gene expression of PPARG and RUNX2, respectively but also HIF-1α. Furthermore, the enhancement of osteogenesis under hypoxia could be mimicked under normoxic conditions by so called “chemical hypoxia” as induced by DFX or DMOG (**Fig. 4**). We also demonstrate here that the hypoxia-mediated increase in osteogenic differentiation and decrease in adipogenic differentiation could be reversed by shRNA against HIF-1α (**Fig. 5**). Of note, HIF-1α in MSCs and other cells mediates the cellular adaption to hypoxia in order to ensure the function of the cells in hypoxic areas through the readjustment of the cellular bioenergetics [7], [8]. Therefore, we conclude from our data that the hypoxic nature of the fracture site facilitates the differentiation of MSCs towards osteoblasts, thereby inhibiting adipogenesis, in order to guarantee the initiation of proper bone regeneration in a HIF-1 dependent manner.

This competitive effect of hypoxia on human MSCs has not been reported previously. However, the investigations on the effect of hypoxia/HIF on human MSC maintenance and differentiation published by other authors show conflicting results.

Focusing on the effect of hypoxia alone, Lavrentieva et al. (2010) reported that hypoxia facilitates a switch from aerobic to anaerobic metabolism that we could confirm by analyzing the gene expression of the glycolytic genes glucose transporter 1 (*GLUT1*), lactate dehydrogenase A (*LDHA*), and phosphoglycerate kinase 1(*PGK1*) [25]. Moreover, Holzwarth et al. (2010) also showed in accordance with our data that low oxygen levels stabilize the immunophenotype of human MSCs [11]. Furthermore, Tamama et al. (2011) and Tsai et al. (2011) very recently demonstrated that hypoxia enhances the proliferation and colony formation thus increasing expansion efficiency of human MSCs [14], [26]. Summarizing the data it has been postulated that oxygen tension is an important regulator in the determination of cell fate and for the maintenance of MSCs in an undifferentiated state, which is supported by our findings [6].

When focusing on the differentiation capability of human MSCs under pathophysiologic conditions, in the case of osteogenic differentiation, hypoxic conditions were shown to reduce the capability of MSCs to differentiate into osteoblasts and into bone marrow-isolated adult multilineage inducible (MIAMI) cells [9], [10], [26], [27], [28]. On the other hand, hypoxia and/or HIF has been reported to promote osteogenesis [29], [30], [31]. The latter data support in part our findings.

With regard to adipogenesis our data suggest that hypoxic condition decreases adipogenic differentiation in a HIF-1-dependent manner. These data were in contrast to the findings of Tsai et al. (2011) as given above [14] but were at least in part supported by Holzwarth et al. (2010) and Tamama et al. (2011) [11], [26]. In the latter report it was shown that hypoxic condition promotes MSC self-renewal through preserving colony forming early progenitors and maintaining undifferentiated phenotypes [26]. Therefore, these authors demonstrated that hypoxic conditions reversibly decreased adipogenic differentiation dependent on the activation of unfolded protein response (UPR), but not on HIFs [26].

We assume that possible reasons for these different results include the variety of sources of MSCs (e.g. umbilical cord-derived [25]), the different culture conditions [14], and differences in the experimental design (use of immortalized human MSCs [26]). Consequently, it is difficult to make broad conclusions regarding the role of hypoxia on the biology of MSCs [32]. However, with regard to our defined experimental conditions we can clearly conclude that hypoxia suppresses adipogenesis and promotes osteogenesis of human MSCs in a competitive and HIF-1-dependent manner. We therefore conclude that the hypoxic nature of the fracture hematoma guides the differentiation of MSCs towards osteoblasts, thereby inhibiting adipogenesis, which is crucial for effective bone healing. During this process, the master regulator of the hypoxic cellular response, HIF-1, balances the differentiation decision between osteogenesis and adipogenesis. Consequently, stem cell therapy in combination with chemical induced hypoxia/HIF-1 could be a new approach to improve fracture healing.

## Materials and Methods

### Antibodies

Antibodies for flow cytometric analysis were purchased at Immunotools (CD13, CD34, CD45, all APC labelled), NatuTec/eBiosience (CD44, CD90, CD105, all APC labelled) and BD Biosience, Heidelberg, Germany (CD73 labelled at DRFZ with Cy5). CD14 and CD19 (labelled with Cy5) were obtained from DRFZ.

For immunoblot, rabbit whole serum anti-human HIF-1α and mouse anti-human β-actin antibody were bought from Abcam plc (Cambridge, UK) and Sigma-Aldrich Chemie GmbH (Munich, Germany), respectively.

### Isolation and expansion of human MSCs

Primary human mesenchymal stromal cells (MSCs) were isolated from bone marrow obtained from human donors undergoing total endoprosthesis (TEP) of the hip joint. The donor population was reasonably homogenous (age range 49 to 68 years, equal numbers of males and females, and exclusively hip osteoarthritis as the reason for TEP). All patients were more or less continuously treated with non-steroidal anti-inflammatory drugs (NSAIDS) (for patient information see Tab. 4). Written informed consent was obtained from each donor. All experiments were conducted according to the protocols approved by the Charité University Hospital ethics committee and according to the Helsinki Declaration. MSCs were isolated with a density gradient and re-suspended in complete culture medium. Human MSCs were cultured in Dulbecco's modified Eagle's medium-high glucose (DMEM) containing 10% fetal calf serum (FCS), 50 µM 2-ME (Sigma-Aldrich), 100 units/ml penicillin, and 0.1 mg/ml streptomycin at 37°C in 5% CO_2_ atmosphere. After 3 days, non-adherent cells were removed by medium change. MSCs were expanded as adherent cells. Cells from each donor were cultured separately. Cells were passaged at 70–80% confluence and passages 3–8 were used for experiments.

**Table 4:** Patient information and experiments conducted.

Patient#	Age	Gender	Medication	Disease	Type of experiment
1	56	f	NSAID*	hip OA#	normoxia (FACS, morphology, adipogenic & osteogenic & chondrogenic differentiation)
2	68	m	NSAID*	hip OA#	normoxia (FACS, morphology, adipogenic & osteogenic & chondrogenic differentiation)
3	66	f	NSAID*	hip OA#	normoxia (FACS, morphology, adipogenic & osteogenic differentiation); hypoxia (FACS, morphology, adipogenic & osteogenic differentiation)
4	49	m	NSAID*	hip OA#	normoxia (FACS, morphology, WB§); hypoxia (FACS, morphology, WB§)
5	65	f	NSAID*	hip OA#	normoxia (FACS, morphology, adipogenic & osteogenic differentiation, qPCR, WB§); hypoxia (FACS, morphology, adipogenic & osteogenic differentiation, qPCR, WB§)
6	59	f	NSAID*	hip OA#	normoxia (FACS, morphology, adipogenic & osteogenic differentiation, qPCR, WB§); hypoxia (FACS, morphology, adipogenic & osteogenic differentiation, qPCR, WB§)
7	58	m	NSAID*		
8	62	m	NSAID*	hip OA#	normoxia (adipogenic & osteogenic differentiation, qPCR); hypoxia (adipogenic & osteogenic differentiation, qPCR)
9	65	f	NSAID*	hip OA#	normoxia (FACS, morphology, adipogenic & osteogenic differentiation, qPCR); hypoxia (FACS, morphology, adipogenic & osteogenic differentiation, qPCR)
10	54	f	NSAID*	hip OA#	normoxia (adipogenic & osteogenic differentiation); hypoxia (adipogenic & osteogenic differentiation)
11	57	m	NSAID*	hip OA#	normoxia (adipogenic & osteogenic differentiation, qPCR); hypoxia (adipogenic & osteogenic differentiation, qPCR)
12	59	f	NSAID*	hip OA#	HIF-knock-down (WB§, adipogenic & osteogenic differentiation)
13	60	m	NSAID*	hip OA#	HIF-knock-down (WB§, adipogenic & osteogenic differentiation)
14	54	m	NSAID*	hip OA#	HIF-knock-down (adipogenic & osteogenic differentiation)

* nonsteroidal anti-inflammatory drugs,

# hip osteoarthritis,

§ Western-Blot.

### Characterization of human MSCs (transduced and non-transduced)

Flow cytometric analysis was used for the characterization of surface protein expression pattern shown by the adherent cells. Typical surface marker CD13, CD44, CD73, CD90, CD105, CD14, CD19, CD34 and CD45 were measured according to protocols previously published [33]. Furthermore, cells were tested for their capacity to differentiate into the adipogenic, osteogenic and chondrogenic lineages *in vitro* (for details see below).

### Induction of hypoxia and “chemical hypoxia”

Human MSCs (transduced and non-transduced) were incubated in a hypoxic chamber (Binder) at 5% CO_2_ level and less than 2% O_2_, balanced with N_2_. Normoxic controls were incubated at 5% CO_2_ in a humidified atmosphere with ∼18% O_2_. When inducing “chemical hypoxia”, human MSCs were incubated under normoxia in the presence of either 250 µM DFX or 100 µM DMOG. Both substances were added to the incubation medium once weekly for a period of 4 weeks.

### Osteogenic, adipogenic and chondrogenic differentiation of human MSCs (transduced and non-transduced)

Human MSCs (transduced and non-transduced) were plated in 6-wells at a density of 10000 cells/cm^2^ for osteogenic and adipogenic differentiation, or at a density of 25000 cells in a pellet culture in a 15 ml polypropylene conical tube for chondrogenic differentiation in a micromass culture. After 24 h, medium was changed to conditioned medium (CM) in order to induce differentiation. In the case of differentiation in the osteogenic linage, DMEM was supplemented with 10 mM β-glycerophosphate, 10 nM dexamethasone and 0.1 mM L-ascorbic acid-2-phosphate. For differentiation of MSCs to adipocytes, 10 µg/ml insulin, 0.2 mM indomethacine, 1 µM dexamethasone and 0.5 mM 3-isobutyl-1-methyl-xanthine was used to supplement DMEM. For chondrogenesis, NH Chondrodiff Medium (Miltenyi Biotec) was used. Undifferentiated MSCs were maintained in control medium, consisting of DMEM supplemented with 10% FCS, penicillin (100 U/ml) and streptomycin (0.1 mg/ml). Medium was replaced every 4 days (chondrogenic) or weekly (osteogenic and adipogenic). Differentiation was terminated after 28 days and assessed by specific staining as described below.

### Oil-Red-O staining of differentiated human MSCs

Cells undergoing adipogenic differentiation were stained with Oil-Red-O dye to visualize lipid droplet accumulation. Medium was aspirated and cells were washed with PBS followed by fixation with 2% neutral buffered formalin for 20 minutes. Then cells were first washed with distilled water and again with 60% isopropanol. A fresh 60% Oil-Red-O working solution was prepared from a stock solution (0.5 g Oil-Red-O in 100 mL isopropanol), left for 10 minutes and filtered through a 0.25 µm syringe filter. The working solution is not stable and has to be used within one day. Cells were stained with the working solution for 15 min, washed with 60% isopropanol and as second with distilled water. Imaging was performed at room temperature by microscopy.

### Von-Kossa staining of differentiated human MSCs

Cells undergoing osteogenic differentiation were stained with von-Kossa dye to visualize calcium deposits, which were replaced by silver. Medium was aspirated and cells were washed with PBS, followed by two washes using distilled water. Cells were fixed with 2% neutral buffered formalin for 20 minutes, and then incubated with 1% silver nitrate solution (w/v) for 60 min under a bright light bulb. Subsequently cells were washed twice with distilled water and then incubated with 5% sodium thiosulfate solution (w/v) for 5 minutes to remove non-reacted silver. Afterwards the cells were washed with distilled water. Imaging was performed at room temperature by microscopy.

### Alzian blue staining of differentiated human MSCs

After chondrogenic differentiation, the medium was removed and the micromass culture pellet was washed with PBS. The pellet was embedded in cryomedium and deep frozen at −80°C. After 2 days, tissue sections were generated and transferred onto microscope slides and either stored at −20°C or directly stained. For the staining, the tissue sections were fixed with 2% neutral buffered formalin for 20 minutes, washed and stained with alzian blue solution for 30 minutes. Staining was followed by washing with tap water and rinsing with distilled water. The stained tissue sections were covered with liquid mounting medium and a coverslip. The tissue sections were either stored at −20°C or assessed directly by microscopy.

### Imaging of differentiated human MSCs

Imaging was performed at room temperature with a microscope (LEITZ DM IL, Leica) equipped with Zeiss A-Plan 106 (NA 0.25) and LD A-Plan 326 (0.40) objectives. Images were captured by a Nikon E4500 camera. Photoshop software (Adobe Systems Inc.) was used to adjust levels and colour balance.

### Quantitative PCR of selected genes of expanded and/or differentiated human MCSs

The cDNAs were synthesized by reverse transcription using Sensiscript® Reverse Transcription Reagents (Qiagen, Darmstadt, Germany). Quantitative PCR (qPCR) was carried out using the LightCycler® Fast Start DNA Master SYBR® Green I Kit (ROCHE Diagnostics - Applied Science, Mannheim, Germany). Data were normalized to the expression of β-actin (*ACTB*). All primers used were obtained from TIB Molbiol (Berlin, Germany; **Tab. 5**).

**Table 5:** Primersets used.

Gene symbol	Gene name	Gene function	Primerset
**ACTB**	beta-actin	structural houskeeper	gACAggATgCAgAAggAgATCACT; TgATCCACATCTgCTggAAggT
**GLUT1**	glucose transporter 1	glucose transport	ACgCTCTgATCCCTCTCAgT; gCAgTACACACCgATgATgAAg
**HIF1A**	hypoxia-inducible factor 1, alpha subunit	transcription factor	CCATTAgAAAgCAgTTCCgC; TgggTAggAgATggAgATgC;
**LDHA**	lactate dehydrogenase A	glycolysis enzyme	ACCCAgTTTCCACCATgATT; CCCAAAATgCAAggAACACT;
**PGK1**	phosphoglycerate kinase 1	glycolysis enzyme	ATggATgAggTggTgAAAgC; CAgTgCTCACATggCTgACT;
**VEGFA**	vascular endothelial growth factor A	pro-angiogenic factor	AgCCTTgCCTTgCTgCTCTA; gTgCTggCCTTggTgAgg.
**PPARG**	Peroxisome proliferator-activated receptor gamma	transcription factor (marker of adipogenesis)	CCCAggTTTgCTgAATgTgAAg; gAAgggAAATgTTggCAgTgg
**RUNX2**	Runt-related transcription factor 2	transcription factor (marker of osteogenesis)	AggTACCAgATgggACTgTg; TCgTTgAACCTTgCTACTTgg

### Immunoblot of HIF-1α and ACTB

Human MSC were lysed after 0 h, 24 h, 72 h, and 2 weeks of incubation under normoxia or hypoxia. Nuclei were prepared using the Nuclear Extract Kit from Active Motif, according to the manufacturer's instructions. For immunodetection of proteins, 10 µg of nuclear fractions were separated by SDS-PAGE and blotted onto PVDF membranes (Millipore). Blotted proteins were analyzed as indicated and visualized by enzymatic chemiluminescence (Amersham Biosciences).

### Lentiviral based shRNA mediated knockdown of HIF-1α

Based on the pLentiLox 3.7 (Addgene plasmid 11795), shRNA constructs were generated by subcloning short hairpin oligo nucleotides (**pLL-scr** 5′-*T gCTATCgAgAAgATCAgCC TTCAAgAgA ggCTgATCTTCTTTAgC TTTTTTC*-3′; **pLL-HIFsh1** 5′-*T CCgCTggAgACACAATCATAT TTCAAgAgA ATATgATTgTgTCTCCAgCgg TTTTTTC* -3′; **pLL-HIFsh2** 5′-*T CCAgTTATgATTgTgAAgTTA TTCAAgAgA TAACTTCACAATCATAACTgg TTTTTTC*-3′) as previously described [34]. Lentiviral stocks were obtained by calcium phosphate co-transfection of HEK293 cells (ATCC) with the lentiviral packaging plasmids pVSVG and pPAX2. The medium was replaced after 4 h and viral supernatants were collected 48–72 h later. Human MSCs were infected by 90 min centrifugation at 700 g at 37°C with viral supernatants and 8 µg/ml polybrene (Sigma-Aldrich), followed by replacement of the viral supernatant with fresh full supplemented culture medium after 4 h. shRNA construct containing cells were analyzed for co-expressed GFP using flow cytometry. Transfected MSC with a frequency of >90% GFP positive cells were used for osteogenic and adipogenic differentiation.

### Statistical analysis

Data shown are reported as the mean ± SD of at least three independently performed experiments. Differences between normally distributed groups were compared using the Student's t test. Statistical significance was considered when p<0.05 (*** p<0.001; ** p<0.01; * p<0.05).
